# Improving care for elderly patients living with polypharmacy: protocol for a pragmatic cluster randomized trial in community-based primary care practices in Canada

**DOI:** 10.1186/s13012-019-0904-4

**Published:** 2019-06-06

**Authors:** M. Greiver, S. Dahrouge, P. O’Brien, D. Manca, M. T. Lussier, J. Wang, F. Burge, M. Grandy, A. Singer, M. Twohig, R. Moineddin, S. Kalia, B. Aliarzadeh, N. Ivers, S. Garies, J. P. Turner, B. Farrell

**Affiliations:** 1 North York General Hospital, 4001 Leslie Street, LE-140, Toronto, Ontario M2K 1E1 Canada; 2 Department of Family and Community Medicine, Faculty of Medicine, University of Toronto, 4001 Leslie Street, LE-140, Toronto, Ontario M2K 1E1 Canada; 3 Department of Family Medicine, University of Ottawa, 85 Primrose Avenue, Ottawa, Ontario K1R 6M1 Canada; 4 Bruyère Research Institute, 43 Bruyère Street, Ottawa, Ontario K1N 5C8 Canada; 5 Department of Family Medicine, University of Alberta, 8303 - 112 Street NW, 610 University Terrace, Edmonton, Alberta T6G 2T4 Canada; 6 Department of Family Medicine and Emergency Medicine, University of Montreal, 1755 René Laennec, Bureau DS-079, Laval, Québec H7M3L9 Canada; 7 Department of Family Medicine, Dalhousie University, 8F, 8525 Abbie J Lane Building, 5909 Veterans’ Memorial Lane, Halifax, Nova Scotia B3H 2E2 Canada; 8 Department of Family Medicine, University of Manitoba, D009 – 780 Bannatyne Ave, Winnipeg, Manitoba R3T 2N2 Canada; 9 Family Practice Health Centre and Women’s College Research Institute, Women’s College Hospital, 76 Grenville Street, Toronto, Ontario M5S 1B2 Canada; 10 Department of family Medicine, Cumming School of Medicine, University of Calgary, G012 Health Sciences Centre, 3330 Hospital Drive NW, Calgary, Alberta T2N 4N1 Canada; 11 Faculty of Pharmacy, University of Montreal, 2900 Edouard Montpetit Boulevard, Montreal, Quebec H3T 1J4 Canada; 12 Centre de Recherche, Institut Universitaire de Geriatrie de Montreal, Montreal, Canada; 13 School of Pharmacy, University of Waterloo, Waterloo, Canada; 14 ICES, Toronto, Canada; 15 Institute of Health Policy, Management and Evaluation, University of Toronto, 500 University Avenue, Toronto, Ontario M5G 1V7 Canada

**Keywords:** Polypharmacy, Inappropriate prescribing, Aged, Primary health care, Quality improvement, Electronic health records, Social facilitation, Clinical trials, randomized

## Abstract

**Background:**

Elders living with polypharmacy may be taking medications that do not benefit them. Polypharmacy can be associated with elevated risks of poor health, reduced quality of life, high care costs, and persistently complex care needs. While many medications could be problematic, this project targets medications that should be deprescribed for most elders and for which guidelines and evidence-based deprescribing tools are available. These are termed potentially inappropriate prescriptions (PIPs) and are as follows: proton pump inhibitors, benzodiazepines, antipsychotics, and sulfonylureas. Implementation strategies for deprescribing PIPs in complex older patient populations are needed.

**Methods:**

This will be a pragmatic cluster randomized controlled trial in community-based primary care practices across Canada. Eligible practices provide comprehensive primary care and have at least one physician that consents to participate. Community-dwelling patients aged 65 years and older with ten or more unique medication prescriptions in the past year will be included. The objective is to assess whether the intervention reduces targeted PIPs for these patients compared with usual care. The intervention, Structured Process Informed by Data, Evidence and Research (SPIDER), is a collaboration between quality improvement (QI) and research programs. Primary care teams will form interprofessional Learning Collaboratives and work with QI coaches to review electronic medical record data provided by their regional Practice Based Research Networks (PBRNs), identify areas of improvement, and develop and implement changes. The study will be tested for feasibility in three PBRNs (Toronto, Montreal, and Edmonton) using prospective single-arm mixed methods. Findings will then guide a pragmatic cluster randomized controlled trial in five PBRNs (Calgary, Winnipeg, Ottawa, Montreal, and Halifax). Seven practices per PBRN will be recruited for each arm. The analysis will be by intention to treat. Ten percent of patients who have at least one PIP at baseline will be randomly selected to participate in the assessment of patient experience and self-reported outcomes. Qualitative methods will be used to explore patient and physician experience and evaluate SPIDER’s processes.

**Conclusion:**

We are testing SPIDER in a primary care population with complex care needs. This could provide a widely applicable model for care improvement.

**Trial registration:**

Clinicaltrials.gov NCT03689049; registered September 28, 2018

**Electronic supplementary material:**

The online version of this article (10.1186/s13012-019-0904-4) contains supplementary material, which is available to authorized users.

Contributions to the literature

- Older patients taking many medications are complex and can be costly for the healthcare system; they often continue to be so over time.
- Stopping medications that may be harmful could improve health for these patients and reduce their healthcare costs.
- This study provides information on complex older patients prescribed ten or more different medications back to their family physician.
- We support the practices by providing tools, collaborative learning sessions, and practice coaching to help them and their patients stop medications if beneficial.
- We will measure whether the data and support resulted in positive changes, compared to family practices not receiving this type of support.

## Background

### Complex patients

In Canada and the USA, 5% of the population incur approximately two thirds of healthcare costs and are identified as patients with complex care needs [1–7]. These individuals receive care in multiple settings and from different healthcare providers [8], making care coordination and integration challenging and resulting in less than optimal care and patient experiences [9, 10]. Strategies to improve care for complex patients are needed [10].

However, complex patients represent different population segments and require approaches that focus on targeted needs. Some patients have elevated care needs related to end-of-life [11, 12]. Others, such as accident victims and transplant recipients, have an acute event requiring intensive resources for a limited time [11]. Only 15% of individuals incurring costs in the top 5% are expected to remain in that category for three or more consecutive years [7]. Approaches to addressing care gaps in individuals with persistent complex needs could produce significant improvements in patient wellbeing and reduce healthcare costs.

Effective strategies for persistently complex patients include care by multidisciplinary teams, trusting relationships, use of technology, adaptability to local context, and assistance with navigating the healthcare system [8, 10, 13, 14]. However, these strategies have not been widely implemented, resulting in a lack of systematic improvement for complex patient populations [9, 15]. Primary health care is patient-centered, involves longitudinal care, provides system navigation, and maintains trusting ongoing relationships with patients and caregivers; it thus represents an ideal setting for improving health and care for persistently complex patients.

There is therefore a need to implement evidence-based interventions to benefit complex patients; these need to be broadly applicable to routine primary care settings in a variety of contexts and need to address individual behavioral change, organizational change, and structure change [16]. An evaluation of the effect of implementation strategies is needed.

When planning this study, we considered [1] whether there was a population of patients that is persistently complex and could be identified to their primary care provider (PCP) and primary care team for action [2]; whether tools exist to support the implementation of care improvements for this population in routine primary care settings [3]; what existing change strategies could be broadly applicable in primary care settings [4]; whether currently available infrastructure in Canadian primary care could support the feasibility of trial recruitment and management for this issue at scale; and [5] what design features would promote adaptation to each context, sustainability, and spread beyond the project.

### Identification of populations of persistently complex patients

#### Polypharmacy as an indicator of persistent complexity

A member of our team (SD) led a study that identified that the number of medications prescribed to a patient is the single most reliable index of persistent complexity, after age. Individuals 65 years or older taking ten or more medications accounted for virtually all (95.3%) patients found to have costs that were consistently in the upper quartile over the following 3 years. This degree of specificity will allow appropriate care to be directed to a primary care practice population based on overall complexity and costs rather than through targeting single health conditions.

### Potentially inappropriate prescriptions as a target for improvement

Many medications are beneficial and clinically indicated while some may be unnecessary or may cause harm in some circumstances. The latter are termed potentially inappropriate prescriptions (PIPs). There are multiple consensus lists that identify which medications are potentially inappropriate, including Beers [17] and STOPP [18]. The Canadian Deprescribing Network [19] and Choosing Wisely Canada [20] have recommended deprescribing PIPs in older patients. Healthcare providers and researchers have prioritized [21] and developed deprescribing tools for specific medication classes [22–25]. These medication classes are proton pump inhibitors (PPIs), sedative-hypnotics, sulfonylureas in older adults, and antipsychotics for indications other than psychosis in older adults.

Legacy prescribing patterns have been shown to contribute to PIPs in cases where patients no longer have the indication for a medication but may continue to receive prescriptions because of clinical inertia [26, 27]. For example, PPIs are often prescribed beyond the recommended short courses of treatment, with no defined benefit to patients and at significant cost and potential for harm (pneumonia, hip fractures, diarrhea), especially in elders [22]. Other medications are contra-indicated; these include benzodiazepines and other sedative-hypnotics used in the treatment of insomnia, which increase the risk of falls, confusion, and hospital admissions [19, 28, 29]; sulfonylureas, which have an increased risk of hypoglycemia in older adults compared to other antihyperglycemic medication classes [23]; and antipsychotics, which increase the risk of stroke and mortality in older adults [30, 31].

“Deprescribing” is the planned and supervised dose reduction or stopping of medication which may be causing harm or no longer be of benefit [32]. There is increasing awareness of the importance of deprescribing in the management of complex patients: several trials and systematic reviews have shown that structured deprescribing is a promising approach to reducing PIPs, including PPIs, benzodiazepines, and sulfonylureas [33, 34–37]. Reviews have also reported some reductions in mortality [38] and improvement in quality of life [39].

#### Tools and processes for deprescribing

Deprescribing is a complex process [40, 41]. Evidence-based guidelines and tools have been created to assist healthcare providers and patients in the deprescribing process [22–25, 42]. Randomized controlled trials (RCTs) have shown that an interprofessional approach, including evaluation by a pharmacist, has improved the appropriateness of medications [43, 44]. The use of benzodiazepines in elders has been reduced through direct education [34]. Patient involvement in a shared decision-making process is essential [41] and is likely to enhance the impact on survival [38]. Communication between the clinician and the patient should evolve from discussions about compliance to dialogs focusing on ensuring that risks and benefit are appropriately communicated and that the patient’s decisions are aligned with their care goals [45, 46]. Involving a pharmacist in the process improves effectiveness and increases patient empowerment and understanding [37, 47]. Appropriately informed community-dwelling seniors in Canada have expressed a preference for reducing the number of medications they take [48, 49]. Finally, effective communication with clinicians outside of primary care will be important to ensure that patients do not get confusing or contradictory messages about their medications from different providers [48].

Thus, a persistently complex population can be readily identified for PCPs; target medication classes and evidence-based deprescribing tools exist to improve care for that population. The next step is to devise implementation strategies focused on deprescribing PIPs for our target population.

### Implementation and change strategies

#### Structured process

Our implementation strategy, the *S*tructured *P*rocess *I*nformed by *D*ata, *E*vidence and *R*esearch (SPIDER), has been designed to help primary care practices optimize their management of patients with complex care needs by combining several evidence-based methods and leveraging existing quality improvement (QI) capacity, partnerships in primary care, and electronic medical record (EMR) evaluation capacity.

##### Quality improvement methods

An important area of practice transformation is systematic improvement in the quality of patient care [50, 51]. The QI Model for Improvement emphasizes a structured approach: define goals, decide on and implement the changes needed, and measure outcomes [52]. QI focuses on rapid-cycle tests of change in a local context while also emphasizing sustainability and spread of effective change within and across healthcare systems [53, 54]. Several Canadian provinces are training healthcare professionals in QI methods to address health quality gaps [55, 56].

##### The Institute for Healthcare Improvement Breakthrough Series model

There is a vast literature that explains why changes, including QI efforts, succeed or fail [57–62]. Common reasons for failure include lack of focus, negative emotions as the project rolls out, lack of influence of the QI team, uncommitted leadership, and lack of cooperation between key stakeholders [63].

We address these challenges with a network of QI Collaboratives that builds on the Institute for Healthcare Improvement (IHI) Breakthrough Series model [64]. The IHI model has four steps [1]: bringing together practices committed to QI to identify a care gap that they agree to be a priority [2], engaging content experts and developing a learning information package that highlights the importance of the care gap and the opportunities for change [3], conducting a learning session during which the practices learn about the challenges and select the change(s) best suited for their context, and [4] implementing the selected changes with ongoing Plan-Do-Study-Act (PDSA) cycles and regular communication. Additional file 1 provides more details about the model. The four steps are a core feature of the SPIDER intervention; they can be adapted to fit each region’s context and resources.

##### Audit and feedback

Audit (measuring quality of care and comparing this against agreed-upon standards of practice) and feedback (delivery of the results to healthcare professionals and/or administrators) allows practices to establish priorities for their population [65–67]. High-performing health systems tend to feature audit and feedback (A&F) [65, 68] as an evidence-based, scalable, and relatively inexpensive strategy to encourage uptake of best practices. We intend to use best practices in A&F as defined in the literature [68].

An example of practice feedback is provided in Additional file 2.

##### Practice coaching

Practice coaching (which has also been called practice facilitation) is an essential element of QI efforts; without it, practice members are less likely to adopt the changes required to optimize care delivery [69–72]. A recent systematic review found that coaching was effective for QI [69]. Coaches bring knowledge about change management and can assist practices to make the desired changes; they help practices develop skills and organize their approach to QI, provide QI tools and expertise, and help to troubleshoot challenges or barriers [72]. Tailoring to the context and focusing on the processes and organization of care are more important than content knowledge [69]. The greater the support provided by practice coaches, the larger the care improvements [69]. Quebec and Ontario have recognized the importance of practice facilitators or coaches, and are supporting their implementation across their regions [73–75].

##### The enhanced Learning Collaborative

**Fig. 1:**
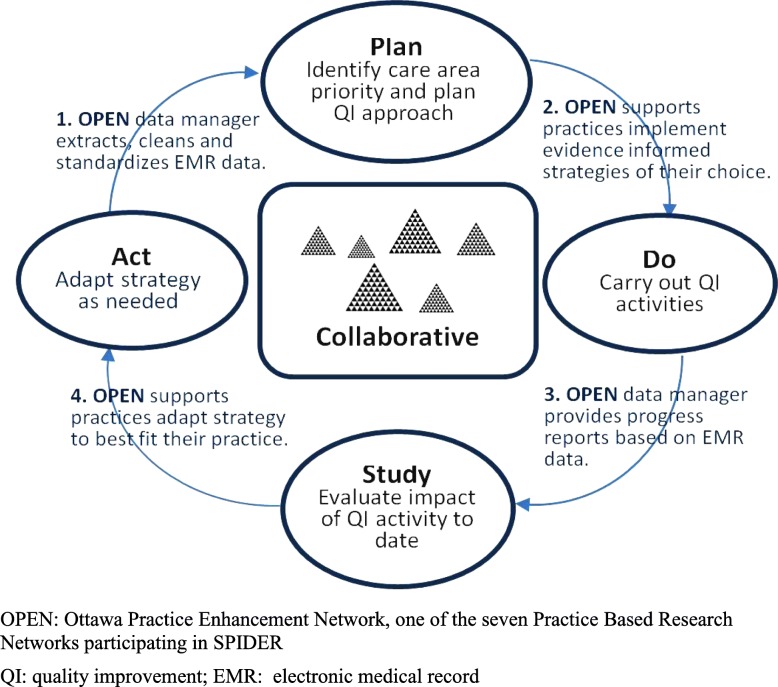
Plan-Do-Study-Act cycle supported by SPIDER. OPEN Ottawa Practice Enhancement Network, one of the seven Practice Based Research Networks participating in SPIDER. QI quality improvement, EMR electronic medical record

SPIDER combines the IHI Learning Collaborative with additional elements, including EMR data for audit and feedback and practice coaching. Figure 1 outlines a Plan-Do-Study-Act cycle supported by SPIDER.

### Feasibility assessment: current infrastructure

#### Regional Practice Based Research Networks

Practice transformation through enhancement of communication and collaboration within and across practice teams requires deep knowledge about primary care in each context. Regional Practice Based Research Networks (PBRNs) designed to facilitate communication, collaboration, and support for innovations already exist in Canadian primary care [76, 77]. A key activity for these regional networks is to improve the quality of care delivered in the member practices. The PBRN movement is growing internationally [78, 79], and the networks are conducting an increasing number of projects in primary care [80]. The College of Family Physicians of Canada, as part of its *Blueprint for Family Medicine Research Success*, is committed to “support[ing] the development and growth of primary care practice-based research networks” [81]. PBRNs collaborate across Canada and the USA, enabling studies across multiple jurisdictions [81–83].

#### Collaboration between QI and research

The traditional distinctions between research and QI in academic centers have sometimes been barriers to conducting collaborative projects [76], but the need for their intersection is greater than ever. This study deliberately spans boundaries between the two approaches. We aim to leverage relationships that already exist at the local and regional level between practice opinion leaders, PBRNs, QI experts, and researchers to strengthen local and regional efforts. Practice coaches supporting practices will meet periodically to discuss issues encountered in implementation and solve the problems; modifications will be made as barriers and early, emerging local data and feasibility issues are encountered.

SPIDER combines QI and research in each region. It leverages EMR data in each PBRN for QI activities while building capacity for QI for family physicians and primary care teams affiliated with their Department of Family Medicine in each region.

#### Availability of validated EMR data

Validated, comparable data is essential to drive QI efforts. It allows practices to identify their performance gaps, compare their performance with external benchmarks such as regional or national averages, set and track care improvement goals, and inform PDSA cycles, the foundational process of QI efforts. Most Canadian PBRNs (including all PBRNs participating in this study) are members of the Canadian Primary Care Sentinel Surveillance Network (CPCSSN) [82]. CPCSSN is a national multi-PBRN organization that works with over 1200 family physicians and nurse practitioners. De-identified patient information is extracted from the EMR systems of multiple vendors, cleaned and standardized by CPCSSN member networks, providing it for research and QI [84, 85]. Data can also be returned to providers, making it accessible to primary care teams [86]. Data returned to practices can be re-identified at the site of care, so that lists of patients to be invited for QI or research can be generated for the practices where these patients receive care [86, 87]. CPCSSN’s database contains the de-identified medical records of nearly 1.8 million patients from primary care clinics affiliated with 12 PBRNs across Canada [77, 85], including the seven participating in SPIDER. CPCSSN has research ethics approvals from all of its sites and from Health Canada to conduct its work [88].

All CPCSSN PBRN members have an existing ethics approval that allows de-identified patient data to be used for research.

### Adaptation, sustainability, and spread

#### Local adaptation

**“**Reinvention” is the extent to which an intervention can be modified to fit the organization and local context as it is implemented [57, 62]. Interventions that cannot be modified or reinvented to fit context are less likely to be implemented [89].

There will likely be co-evolution (adaptation of the intervention as well as adaptation of the team) during implementation of changes [58]. A study of the implementation of quality standards in business organizations found that improvements in performance were correlated with both successful reinvention of the innovation and the ability of the organization to transform itself to take advantage of the innovation [90].

Because of this complexity, some aspects of implementation will always be unpredictable and will depend on local context. Therefore, providing core specifications, while at the same time outlining adaptable elements and building capacity for reinvention by practices, are fundamental components of SPIDER [91].

#### Sustainability and spread

Successful changes are vulnerable to challenges of spread and sustainability. Fewer than 40% of initiatives to improve health care move from the pilot project stage to sustained implementation that spreads to more than one area of an organization, much less outside that organization [92]. Medical Associations have called for coordinated efforts to strengthen primary care’s ability to spread QI activities responsive to local needs [93, 94].

SPIDER offers a strategy to support practices as they address regional priorities by allowing implementation to be adapted to the local context; individual practice changes can be tailored to the specific profile of practice members. Sustainability is enhanced through establishing changes that are acceptable to the practice members, have a manageable degree of disruption on every day functioning, and can be embedded in daily practice [95]. Spread needs to be informed by experience across various regions. The intervention must have enough reproducible elements to enable replication in additional settings yet be sufficiently adaptable for each context.

### Objectives

The primary objective of this study is to evaluate the impact of the SPIDER model compared to usual care in the management of PIPs across the quadruple aim: health, care experience, value, and care provider work experience [96, 97]. We are not aware of large-scale projects or activities targeting the population of interest for SPIDER in the Canadian context.

The primary study outcome is to determine whether the SPIDER model reduces PIPs for patients aged 65 and older with polypharmacy, when compared to usual care. Secondary outcomes include assessing SPIDER’s effect on patient health-related quality of life, costs, and provider experience.

### Study design

The study has two phases: feasibility and randomized controlled trial (RCT).

The feasibility of SPIDER will be studied in three Canadian regions (Toronto, Edmonton, and Montreal) using a single-arm, prospective, explanatory mixed methods approach. We will then adapt and apply what we learn from the first phase to a pragmatic cluster RCT [98] with two parallel arms (usual care control, intervention) involving five Canadian regions (Winnipeg, Halifax, Calgary, Ottawa, and Montreal). The pragmatic design (minimal patient selection criteria and tailoring of the intervention to the practice setting) enhances generalizability to other patients in diverse primary care practices. As such, the study will investigate the “real world” impact of the intervention and enhance its generalizability [99, 100]. The trial is designed to test for superiority of the SPIDER approach. Randomization will be stratified by region, with a 1:1 allocation.

We will use an open cohort design where patients identified as belonging to the target population at baseline are followed prospectively over time, regardless of the number of medications prescribed in these subsequent periods. New patients meeting the inclusion criteria identified during the follow-up periods are entered into the cohort.

Patients will be excluded from the evaluation if their EMR indicates that they are deceased or transferred out of the practice; the date entered in the EMR will be used for exclusion. If at a point in time a member of the cohort is found to have no new prescriptions issued in the EMR in the prior 12 months, that individual will be deemed to be either deceased or transferred out of the practice and will be excluded from the evaluation. At each evaluation period, new individuals meeting the target population profile may enter the cohort.

We are using the SPIRIT statement to report the protocol [101], and the intervention is being described using the TIDieR checklist [102].

## Methods

### Study settings

Seven Canadian Primary Care PBRNs that are members of the CPCSSN across five provinces are involved in the study.

#### Canadian setting

The Canadian healthcare system includes universal coverage for all medically necessary procedures and visits provided by physicians or in hospital under the Canada Health Act [103]. Because health care is largely managed within each province, differences in primary care organizations exist across Canada. These include availability of interprofessional care and differences in physician remuneration and public coverage of medications for individuals age 65 or over.

### Eligibility criteria

Practices are eligible if team members provide comprehensive family medicine in an office setting regardless of practice funding model (fee for service, capitation, or salaried) or team composition and have at least one provider consenting to participate. PCPs (family physicians or nurse practitioners) are required to offer comprehensive primary care services to community members, contribute EMR data to CPCSSN, and allow the research team to survey and/or interview selected patients from their practice. The target patient population consists of individuals ≥ 65 years of age receiving care from a participating provider, with more than one office visit during the past 2 years, and had received ≥ 10 different prescription medications in the past year as indicated in the EMR.

### Intervention

Practices randomized to the intervention will identify one practice champion and a team of practice administrator(s) and clinicians to participate in the 12-month Collaborative and Structured Process described earlier (see “Implementation and change strategies” section). Practices are encouraged to engage their practice pharmacist, where one is available, or a community pharmacist in the initiative.

We will generate a list of anonymized patient codes identifying patients meeting the eligibility criteria for each provider which can be re-identified at the clinical site. Medications in CPCSSN are coded using the Word Health Organization’s Anatomical-Therapeutic-Chemical (ATC) classification [104]. ATC codes used to determine PIP classes in this study are shown in Additional file 3. Each regional PBRN will generate the list using the same algorithms and will provide the information to participants.

Practices participating in the intervention will form a Learning Collaborative for each region. The elements that can be adapted are the setting for the learning sessions (single day vs two half days for the initial session; in person vs virtual to reflect geography and availability of healthcare providers), composition of healthcare team members to reflect local context, and the extent of availability of practice coaches to reflect funding for QI in each region.

### Outcomes

#### Feasibility phase

We will evaluate the feasibility of the SPIDER approach across eight dimensions: acceptability to stakeholders, demand, implementation, adaptation, integration, practicality, and efficacy of the SPIDER model [105, 109, 110], as well as evaluating participation and approaches to outcome measurement (see Table 1).

**Table 1:** Evaluation of feasibility

Acceptability	
Patients	Veterans Affairs multidimensional survey [59] capturing five dimensions related to polypharmacy and deprescribing: “medication concerns,” “provider knowledge,” “interest in stopping medicines,” “unimportance of medicines” and “patient involvement in decision-making.” [106] Supplemented with interview questions on experience with the process, symptoms (improvements/new), relationship with PCP, empowerment, and care coordination dimensions identified in previous qualitative research as pertinent [41, 106–108]. These will be administered at 12 months
Providers	Semi-structured interviews with selected providers based on the Theoretical Framework of Acceptability [109] (Table 2) Focus group and survey based on Organizational Readiness to Change Assessment (ORCA) and Data-Driven Quality Improvement in Primary Care (DQIP)
Demand	Coordinator’s log: enrolment and retention of practices and providers
Implementation	Coordinator’s log: ability to apply the SPIDER elements as planned Project memoranda: implementation facilitators and barriers; best practices
Adaptation	Coordinator’s log: fidelity to SPIDER process, and extent of change required to accommodate SPIDER to the context
Integration	Extent of effective collaboration across sectors (semi-structured interviews with selected practices)
Practicality	Ability to integrate the process into existing practice (semi-structured interview with selected practices)
Efficacy	Potential for approach to achieve desired outcomes: EMR-based PIPs, survey, patient health-related quality of life [110], emergency room visits and hospitalization where available from administrative data
Evaluation	Ability to consistently extract PIPs across PBRNs and derive the outcome measures Ability to link study participant to health administrative data and extract emergency room data and hospitalization (Ontario) Completeness of surveys (and individual components) by PCPs and patients and participants’ comments on these (e.g., content, clarity, length) Participation of PCP and patients in interviews

#### Randomized control trial phase

This phase of the study evaluates the impact of the SPIDER model across the quadruple aim. A summary of all outcome measures is shown in Table 2.

**Table 2:** Summary of outcomes (RCT phase)

Dimension/measure	Target population	Assessment	Time
Health outcomes			
Primary outcome: PIP prevalence: Absolute reduction in the prevalence of PIPs, defined as # PIP in target population/# patients in target population	All individuals (intervention and control): • Member of the practice of a participating provider; 65 and over; with 10 + prescription in previous 12 months Post-intervention only: All patients identified at pre-intervention, alive, and member of the practice. New patients meeting criteria may enter the cohort	EMR data extraction See operationalizing this indicator in Additional file 4	Baseline: prevalence of PIPs during the 12 months prior to the start of the intervention Post-intervention: prevalence of PIPs during the 12 months following the intervention
Patient prevalence: Absolute reduction in number of patients with at least one PIP	As above	As above	As above
Quality of life	A 10% randomly selected subset of individuals identified at baseline as having at least one PIP (intervention and control)	EuroQOL-5D [111]	Post-intervention
Patient experience			
Medication-related experience	Same as above	Survey adapted from the Veterans Affairs multidimensional survey [59, 106]	Post-intervention
Experience with care	Same as above but in the intervention arm only	Semi-structured interviews	Post-intervention
Provider experience			
Experience in Collaborative and in deprescribing PIPs	All PCPs in the intervention arm only will be invited	Survey adapted from existing tools [112, 113]	Post-intervention
Experience in Collaborative and in deprescribing PIPs	At least one PCP from each practice in the intervention arm only will be invited	Focus groups	Post-intervention
Costs			
Cost-benefit	All individuals identified at baseline as eligible (intervention and control), in Ontario only	Health administrative data	Post-intervention, end of follow-up period (12 months after intervention completed)
Estimate of costs of medication, delivering the enhanced QI program (materials, management costs for the program, EMR data extraction and analysis), and practice facilitation	All practices and patients in intervention arm only	Program manager logs and records	Post-intervention

##### Potentially inappropriate prescriptions

A PIP is defined as at least one prescription in a class of interest during the period being measured (see Additional file 3). Each class is counted once only during each 12-month period. An individual may therefore only contribute up to 4 PIPs in any one period.

We will also ascertain the prevalence of patients 65 years or older with at least ten unique prescriptions and at least one PIP, the prevalence of each individual PIP class, and the prevalence of patients with each individual PIP class.

Finally, we will evaluate quality of life in a subset of randomly selected patients at the end of the intervention using the EuroQOL-5D [111, 114].

##### Patient experience

A 10% random sample of the patients identified at baseline as having PIPs will be invited to participate in a survey about their medication (both arms) and potentially an interview (intervention arm) to explore elements identified in previous qualitative research as pertinent [41, 106–108].

We will measure patient experience using the Veterans Affairs multidimensional survey capturing five dimensions related to polypharmacy and deprescribing [106].

##### Provider experience

We have adapted existing tools to survey providers about their experience in the Collaborative [112], and their experience and confidence in deprescribing PIPs [113, 115, 116]. We will conduct PCP focus groups in each region to explore the following: experience in the SPIDER Collaborative, appropriateness of guidelines provided, time required and burden, communication and shared decisions with patients and other providers, and confidence in deprescribing.

##### Costs

We will conduct a cost-benefit analysis. We will capture the costs delivering the enhanced QI program (materials, management costs for the program, EMR data extraction and analysis), practice facilitation for the intervention arm, and the overall healthcare costs incurred in the 24 months post-intervention using a previously validated person-centered costing methodology [117, 118].

We will also conduct focus groups with policy makers to understand their views of this approach and how policies could support integration of similar initiatives into primary care.

We will use the Consolidated Framework for Implementation Research (CFIR) to evaluate the implementation of SPIDER [16]. The evaluation will include information about the adaptable elements in each region: types of sessions in the Learning Collaborative (in person, online), availability of interprofessional team members including pharmacists, and extent of availability of practice coaches.

### Participant timeline

**Fig. 2:**
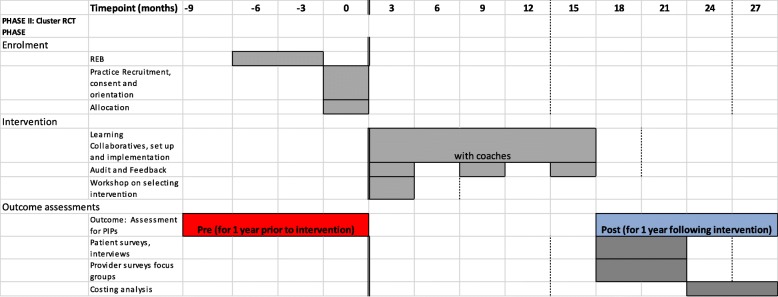
Schedule of enrolment, intervention, and outcome measurements for RCT

An overview of the schedule of enrollment, randomization, interventions, and assessments for the RCT is provided in Fig. 2. The feasibility phase is similar; there is no allocation (single arm), and an assessment of processes and feasibility will occur during and shortly after the intervention. The intervention will begin after allocation, with a workshop, provision of feedback, and introduction of coaches. The Learning Collaborative lasts about 12 months; three additional months have been allocated to account for local context, such as booking room or conducting two separate half day workshops. Periodic Learning sessions will occur during the Learning Collaborative; communication will occur using email or webinars.

Interview focus groups and surveys will be conducted shortly after the end of the Collaborative.

EMR-based outcome data will be extracted for the year prior to the intervention and for the year after the intervention.

### Sample size

#### Sample size calculation

Preliminary analyses conducted on 86 practice sites of the University of Toronto PBRN (UTOPIAN) involving 334 PCPs found the following [1]: an average of 24 patients per PCP had ≥ 10 unique prescriptions in the past year [2]; the prevalence of PIPs in this patient population was PPI 58.1%, benzodiazepine 33.0%, hypoglycemics 1.2%, and antipsychotics 6.9% [3]; 73.1% of patients had ≥ 1 PIP (composite prescription rate) [4]; the mean number of PIPs was 0.987 per patient (standard deviation 0.748); and [5] the intra-cluster (practice) correlation (ICC) was 0.017 using the composite prescription rate as the primary endpoint.

We will recruit about eight practices in each of the three PBRNs participating in the feasibility phase of this study, allowing us to understand various facets of implementing the approach in three geographical regions. The recruitment in the feasibility phase will inform how the approach might need to be tailored to optimize its implementation in the RCT phase.

For the RCT portion, assuming a conservative ICC of 0.05 and two participating providers per practice site (46 patients), 28 practices in each arm are required to detect a mean difference of 15% (from 0.987 to 0.837) in PIPs, allowing for *α* = 0.05 and *β* = 20%. We will recruit 14 practice sites per region (total 70) to allow for attrition. The unit of randomization will be the primary care practice, and analyses will be conducted at the patient level.

### Recruitment

Each PCP must provide written informed consent to be included in the SPIDER trial; consent must be obtained prior to randomization for the cluster RCT.

The leads of the seven PBRNs participating in this study (each is a principal investigator on this study) will recruit the required number of practices for this study. As part of their infrastructure, PBRNs hold regular meetings and communicate with members [119].

We will use purposeful sampling to invite participants (family physicians, interprofessional team members, staff members) in each center to participate in focus groups. We will aim to get maximum variation across profession, sex, within each region.

### Allocation and concealment

Allocation is conducted at the practice site level.

Allocation to either intervention or usual care control will be 1:1, stratified by region and using a computer-generated central randomization schedule. A senior statistician at the Department of Family and Community Medicine, University of Toronto, will oversee the generation of the allocation sequence. Concealment will be maintained by providing the randomized allocation to each region once all practice sites have been recruited for a region.

### Blinding

Blinding of participants is not feasible in pragmatic trials of QI interventions in primary care [120]. The identification of PIPs and medication count (at baseline and end of study) will take place centrally by a data manager blinded to the arm attribution to avoid potential biases in cases where ambiguity about the presence of the drug requires their assessment of additional patient data. Similarly, data cleaning and imputation will be performed centrally by a staff member blinded to the arm attribution.

### Data collection

All physicians participating in the RCT are required to be existing members of the CPCSSN initiative and would therefore have already contributed de-identified EMR data to regional network repositories. The method of EMR data extraction and the approach to processing the data (coding and standardizing) have been previously described [121]. CPCSSN has experience obtaining data elements required for this initiative [77, 85, 122] and generating lists of prescribed medication by time period, including attribution to the class of drug [123–125]. We will therefore be using EMR data routinely collected in primary care and managed by the PBRNs affiliated with CPCSSN for the primary study outcome.

A survey for patients with PIPs will be delivered to patients of study participants in both arms by a research staff member at 12 months.

A short survey will be administered at baseline to both arms asking PCPs and their staff about their practices. PCPs and staff of practices participating in each Collaborative (intervention arm) will be asked to complete an online survey about their experience with the SPIDER process and its impact on their ability to care for patients with complex needs at 12 months after baseline. Interviews of patients and focus groups with providers and policy makers will be conducted only for the intervention arm at 12 months. These will be audiotaped and transcribed verbatim.

### Statistical methods

Descriptive statistics will be used to provide information about physician and patient characteristics, participation rates, polypharmacy, and PIPs. The analysis will be on an intention to treat basis; it will therefore include physicians who have agreed to participate but discontinue their engagement at any point during the study. Outcomes are measured at the patient level; we will use hierarchical modeling with random effect variables and/or generalized estimating equations to account for clustering; intra-cluster correlation coefficients will also be reported. All patients of participating physicians who had been identified at baseline as eligible, regardless of whether they had a PIP at baseline or not will be included in the analysis. The number of PIPs prescribed per patient in the control and intervention arms over a 12-month period following the intervention in each regional network will be compared. A framework of analysis of covariance [126] will be used to adjust for potential confounders such as age, sex, comorbidity, and outcomes.

## Discussion

This study enables the identification of persistently complex patients, elders living with polypharmacy, for their family physicians and primary care teams. The QI intervention uses evidence-based tools and processes to deprescribe medications that have been identified as being potentially inappropriate by highly credible national organizations. It leverages relationships between clinicians, patients, QI experts, and researchers to implement an intervention with multiple evidence-based components, adapt it to multiple settings, and measure its effects. It uses comparable, validated EMR data for patient identification, recruitment, and outcome measurement.

A strength of the study is the identification of patients living with persistent care complexity in primary care in a way that can be actioned by physicians and their care teams. The focus is not on any single disease. As well, the design is pragmatic, reflecting usual care and patients followed in primary care practices, rather than highly selected populations, allowing greater external validity. The intervention chosen for this project uses QI principles and is led and managed by experts in QI. It reflects the principles of a Learning Healthcare System, where “science, informatics, incentives and culture are aligned for continuous improvement and innovation, with best practices seamlessly embedded in the delivery process and new knowledge captured as an integral by-product of the delivery experience.” [127].

There are several limitations to this approach. Data may not be captured completely in EMRs; however, we do not expect any a priori differences between practices randomized to intervention vs usual care control with respect to data entry processes. The sample represents primary care practices that contributed EMR data to CPCSSN, rather than a random sample from the population of all primary care practices. Physicians participating in CPCSSN are slightly younger and more likely to be female compared to the population of physicians who have responded to the National Physician Survey [84].

EMR data entry on patient status may not be timely or completely accurate; it is possible that some patients are no longer in a practice due to admission in long-term care facilities and transfer to another physician or death and that change in status has not been recorded. Physicians in the intervention arm receive data on patients with polypharmacy and may be prompted to review and clean data on patient status, leading to an imbalance between the two arms. To address this, we will ascertain eligible populations in both arms prior to intervention, carry forward all patients initially identified, and limit eligible populations to those with at least one prescription in the EMR during the 12 months following the intervention period.

EMR data for audit and feedback provided through SPIDER incurs delays related to data acquisition and processing. Typically, data reflects care that occurred between 3 and 6 months prior to feedback. The delay is not ideal for feedback, which needs to be timely [68]. CPCSSN data is collected once every 3 months; monthly run charts are preferred for QI. To address this, teams will use their own EMR data for internal rapid-cycle testing and changes. CPCSSN data are provided at the outset and periodically for external comparisons.

We are deliberately allowing local adaptation so that the intervention is applicable to multiple settings and contexts. This improves external validity while adding challenges to intervention fidelity. We are addressing this by testing the intervention in three different regions (Toronto, Edmonton, and Montreal) as part of a feasibility phase, prior to the RCT. This will allow us to test feasibility of fidelity to “core” elements and study implementation of “adaptable” elements.

We have defined core elements for the SPIDER intervention as consisting of a Learning Collaborative, practice coaches, and audit and feedback using validated EMR data. The design does not allow us to determine which of these aspects is effective; each has prior evidence of effectiveness, and we are measuring the cumulative effect of the combined elements. We are allowing flexibility in implementing the IHI model for the Learning Collaborative as appropriate for each context, provided the IHI’s four steps are followed.

## Conclusion

If successful, this trial will provide broadly generalizable evidence that the multi-pronged SPIDER intervention improves care for elders living with polypharmacy by reducing PIPs. Should SPIDER be successful for this population with complex care needs, the approach could be replicated to improve care for a variety of issues facing patients, their physicians, and their primary care teams and organizations. Policy makers could consider whether the elements included in SPIDER (Learning Collaboratives, EMR data for audit and feedback, practice coaching) should be supported as part of high functioning healthcare systems.

## Additional files

Additional file 1: Description of the IHI breakthrough series model and SPIDER intervention (DOCX 201 kb)

Additional file 2: An example of practice feedback (DOCX 389 kb)

Additional file 3: ATC codes used to determine PIP classes in this study (DOCX 22 kb)

Additional file 4: EMR data extraction (PDF 553 kb)

## Abbreviations

A&F: Audit and feedback
CFIR: Consolidated Framework for Implementation Research
CPCSSN: Canadian Primary Care Sentinel Surveillance Network
DQIP: Data-driven Quality Improvement
EMR: Electronic medical record
ICC: Intra-cluster correlation coefficient
IHI: Institute for Healthcare Improvement
ORCA: Organizational Readiness to Change Assessment
PBRNs: Practice Based Research Networks
PCP: Primary care provider
PDSA: Plan-Do-Study-Act
PIPs: Potentially inappropriate prescriptions
PPIs: Proton pump inhibitors
QI: Quality improvement
RCT: Randomized controlled trial
SPIDER: *S*tructured *P*rocess *I*nformed by *D*ata, *E*vidence and *R*esearch
UTOPIAN: University of Toronto Practice-Based Research Network
