# Supplementary material for: Improving care for elderly patients living with polypharmacy: protocol for a pragmatic cluster randomized trial in community-based primary care practices in Canada
Source: Implement Sci. 2019 Jun 6;14:55. doi: 10.1186/s13012-019-0904-4 (PMC6551894; doi:10.1186/s13012-019-0904-4)
Supplement: Supplementary file 1 — Description of the IHI breakthrough series model and SPIDER intervention (DOCX 201 kb) [file 13012_2019_904_MOESM1_ESM.docx]

Description of IHI Breakthrough Series Model and SPIDER Intervention


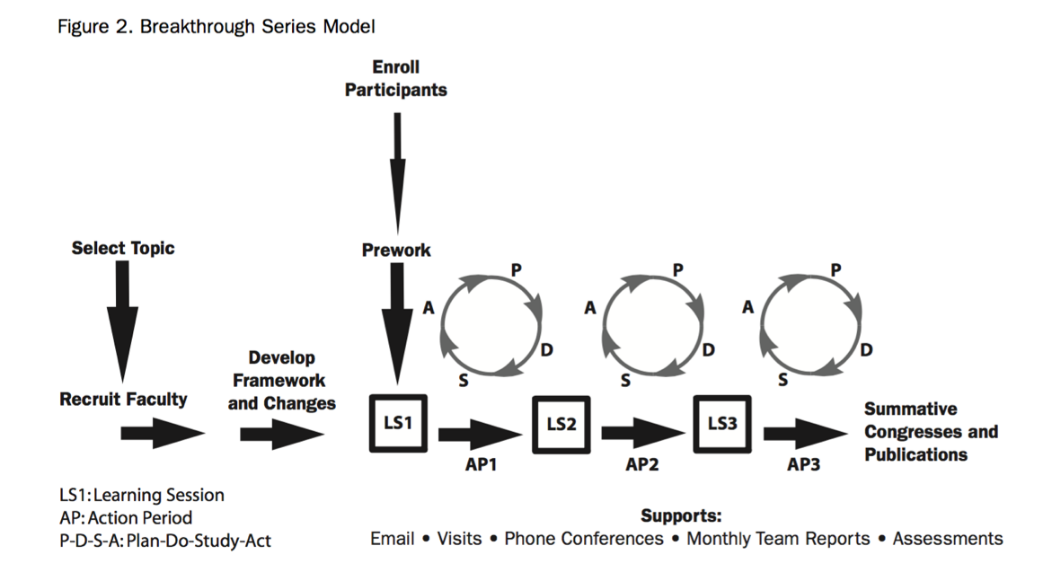


*The Breakthrough Series: IHI’s Collaborative Model for Achieving Breakthrough Improvement.* IHI Innovation Series white paper. Boston: Institute for Healthcare Improvement; 2003. (Available on [www.IHI.org](http://www.ihi.org/) or via <http://www.ihi.org/resources/pages/ihiwhitepapers/thebreakthroughseriesihiscollaborativemodelforachievingbreakthroughimprovement.aspx>

*SPIDER enhances this model as follows: team formation (the “enroll participants” and “recruit faculty” stages of the BTS) leverages pre-existing regional primary care networks; and topic selection, prework, the learning sessions, and the action periods are informed by data from a nationally validated primary care electronic medical record (EMR) dataset*.

The Institute for Healthcare Improvement (IHI) Breakthrough Series (BTS) Learning Collaborative was developed by IHI to support breakthrough improvement (see figure). It is a structure that enables networking and sharing between teams and permits learning between teams and from experts (in both content and process). The BTS is characterized by education-focused, in-person events or learning sessions followed by action periods. Teams come together for learning sessions to learn, network, share, and profile their work. During action periods between the learning sessions, teams work on their quality improvement (QI) project, measuring, analyzing systems, and testing change.

The SPIDER approach builds on this model; the process specific to this study is as follows:

Step 1: Create regional SPIDER QI Collaboratives

In each region, practices designated to the intervention arm of the randomized controlled trial, policy-makers, and patient partners will be brought together to form a QI Collaborative as part of SPIDER. They will work together to shape the approach locally. Each practice will delegate a team to represent their practice population in SPIDER. They will identify one practice champion who will be responsible for leading the initiative in their practice and establishing a team of practice members who will participate in the Collaborative. The practice team may include administrative staff and other health professionals in the practice (e.g., nurse or pharmacist). Regional researchers, practice facilitators, patient partners, experts in QI, policy-makers and content experts (polypharmacy and deprescribing) will round out the Collaborative membership. The three sites (Toronto, Edmonton and Montreal) in which the feasibility phase of the study is being conducted have had considerable experience setting up collaborative QI initiatives.^1-4^

The activities for the partnership will include: applying QI measurement principles, system analysis tools, and change testing methods to enable practice improvement; providing ongoing CPCSSN data from the PBRNs that are relevant to each team’s project; developing a sustainability and spread plan; and preparing a dissemination plan.

The overarching model was discussed with all partners in the Collaborative, including patient partners; the following sections represent the approach that resulted from these discussions.

Step 2: Conduct Audit and Feedback, set goals and plan actions

All members of the Collaborative will participate in an 8-hour workshop (full day or divided as required for local context) during which participants will be provided with data on polypharmacy and potentially inappropriate prescriptions (PIPs). They will review options for addressing the issue and agree on goals and targets. The workshop will address general polypharmacy issues, how these issues relate to various PIPs, and approaches to ameliorating them. Special attention will be given to proton pump inhibitors (PPInhs), benzodiazepines/sedative-hypnotics, sulfonylureas, and antipsychotics because Canadian guidelines and tools for deprescribing are currently available for these types of medications.

**Evidence**: SPIDER will prepare information on the impact of PIPs and compile empirical evidence about various options that have shown promise in reducing PIPs and their estimated impact. The information will cover the health risks associated with PIPs, anticipated benefits and potential unintended consequences of discontinuing a medication, and consequences of PIPs on patient well-being and on various healthcare sectors, to allow all stakeholders to appreciate the relevance of the issue. Each stakeholder group will be invited to contribute to creating the information and encouraged to deliver the information relating to their group (e.g., policy-makers on impact across healthcare sectors; patient partners on impact of patient well-being). Members of CaDeN taking part in this application and our pharmacy team will provide major input.

**Audit and Feedback**: In preparation for this meeting, EMR data from all participating practices will be extracted using the confidential and secure CPCSSN process. Information on the number and type of medications and the prevalence of PIPs will be assessed using centrally developed algorithms. Results of this audit will be provided to each Collaborative (feedback) during the workshop. Collaboratives will receive practice- and regional-level data as well as cross-regional comparative data. Participating practices will receive their own data confidentially.

An example of feedback is provided in Appendix xx.

**Goals Setting**: Participants will now be aware of the current PIP levels and will be informed by scientific knowledge about the potential for improvement. We will apply a facilitated deliberative process to elicit patient priorities and help each Collaborative establish collectively the priorities they choose to address and the goals they want to achieve over the following 12 months. The process will ensure that the voices of all members of the Collaborative are heard.

**Action**: Effective approaches to reducing PIPs are based on (1) provider-targeted education strategies (e.g., increasing awareness, providing guidelines for deprescribing), (2) patient-directed educational strategies (e.g., understanding of potential risks and benefits of being on the medication and of discontinuing it), and (3) pharmacist interventions (e.g., use of medication review and patient pharmacy consultation). Studies using more than one approach may be more effective. We will support each practice team (providers, staff, patient partners) as they select the approach(es) best suited to their context.

**Facilitated Implementation:** Coaches will be assigned to support the process for each participating practice. This will include in-person meetings at the practices, teleconferences, and information sharing as part of SPIDER. There will be flexibility to reflect the needs of practices, as well as time to build the necessary relationships.^5^ Coaches will support practices in doing frequent testing and tracking during the action period. At six months, the regional coordinator will convene a second facilitated collaborative meeting. At that meeting, a progress report based on each practice’s own internal data will be shared, and the members will describe their ideas for change and their challenges and successes with testing, implementation, and overcoming barriers. The meeting will enhance collaboration between members; each group represented will be given an adequate opportunity to contribute, to optimize progress in the following six months and to maximize the potential for success and sustainability.

**Plans for Sustainability**: The regional coordinator will convene a summative congress at 12 months, during which the results of a post-intervention audit and feedback and the results of patient surveys and interviews will be presented. The Collaborative will review these results, discuss their challenges, share their successes, and discuss plans for sustainability and spread.

Practice teams that join a Collaborative will not be charged a fee for participating, but they will be expected to participate in all elements of their Collaborative. This includes attending meetings (in person or virtually), engaging in QI coaching, and participating in the evaluation and dissemination of the Collaborative’s results. Mainpro+ educational credit hours will be provided.

1. Harris SB, Green ME, Brown JB, et al. Impact of a quality improvement program on primary healthcare in Canada: a mixed-method evaluation. *Health Policy.* 2015;119(4):405-416.

2. Quality and Innovation. <http://www.dfcm.utoronto.ca/landing-page/quality-innovation>. Published 2017. Accessed.

3. Grunfeld E, Manca D, Moineddin R, et al. Improving chronic disease prevention and screening in primary care: results of the BETTER pragmatic cluster randomized controlled trial. *BMC Fam Pract.* 2013;14:175.

4. Bareil C, Duhamel F, Lalonde L, et al. Facilitating Implementation of Interprofessional Collaborative Practices Into Primary Care: A Trilogy of Driving Forces. *J Healthc Manag.* 2015;60(4):287-300.

5. Nagykaldi Z, Mold JW, Robinson A, Niebauer L, Ford A. Practice facilitators and practice-based research networks. *J Am Board Fam Med.* 2006;19(5):506-510.
