# Supplementary material for: Improving care for elderly patients living with polypharmacy: protocol for a pragmatic cluster randomized trial in community-based primary care practices in Canada
Source: Implement Sci. 2019 Jun 6;14:55. doi: 10.1186/s13012-019-0904-4 (PMC6551894; doi:10.1186/s13012-019-0904-4)
Supplement: Supplementary file 2 — An example of practice feedback (DOCX 389 kb) [file 13012_2019_904_MOESM2_ESM.docx]

**SPIDER: Structured Process Informed by Data, Evidence and Research**

| Attn: [Practice/Team Name] |
| --- |
| **An Approach to Supporting Primary Care Practices in Optimizing the Management of Patients with Complex Needs** |
| **Practice Feedback Data** |

| **SPIDER Learning Collaborative – Toronto**  **Date** |
| --- |

SPIDER Safer Prescribing for Elders: Overview

# Goal

- **Safer prescribing** for older patients (65+ years) prescribed 10 or more different drugs
- **Focus on deprescribing of 4 medication classes: PPIs, Benzodiazepine, Sulfonylureas, Antipsychotics**
  - There is evidence that safer prescribing for these medications may help Elders. Reducing the dose or eliminating the prescription can improve outcomes for some older patients (Choosing Wisely Canada; Canadian Deprescribing Network)

# Background and Rationale

- More than **one in four** Canadians aged 65 or over has been prescribed **≥10 different medications** in the past year^[[1]](#footnote-1)^.
- In this network (UTOPIAN), each family physician has, on average, **24 older patients** with prescriptions for >10 different medications.
- Too many of these patients are taking **PPIs, Benzodiazepines, Sulfonylureas or Antipsychotics**; discontinuing these may be beneficial to many.
- The risks associated with these medications are significant for Elders. For example
  - PPIs: pneumonia, hip fractures, diarrhea
  - Benzodiazepines: falls, confusion
  - Sulfonylureas: hypoglycemia
  - Antipsychotics: stroke, increased mortality rates
- Benefit vs potential harm for these 4 medication classes **should be reassessed regularly**

# The What and How

There are different strategies you can use to address medication safety. The work can be adjusted to fit into your practice. We can provide assistance with establishing processes best suited to your practice.

- You could start with selected patients or medication-classes, or start by identifying those on the most medication-classes.
- Here are some tools to help you with this work:
  - <https://choosingwiselycanada.org/perspective/ppi-toolkit/>
  - <https://choosingwiselycanada.org/perspective/toolkit-benzos-primary-care/>
  - <https://choosingwiselycanada.org/perspective/antipsychotics-toolkit/>
  - <https://deprescribing.org/wp-content/uploads/2015/11/deprescribing_algorithms2016_AHG_vf-cc-Sept-2016-InDesign.pdf>

My Practice At-a-Glance^[[2]](#footnote-2)^

# Number of Eligible Patients

- My practice as **163** older patients (age ≥ 65) in total
  - Of these, **22** have 10 or more unique medications
- My practice has **13** older patients prescribed at least one of the four targeted medication-classes in a year
  - **7** older patients have a prescription for a PPI
  - **5** older patients have a prescription for a Benzodiazepine
  - **1** older patients have a prescription for an Antipsychotic
  - **2** older patients have a prescription for a Sulfonylurea

# Compared to the Best 10% of UTOPIAN practices

- Compared to the best 10% of UTOPIAN practices, my practice has
  - **higher** % of older patients prescribed 10 or more unique medications
  - **higher** % of older patients on 10^+^ meds prescribed a PPI
  - **higher** % of older patients on 10^+^ meds prescribed a Benzodiazepine
  - **higher** % of older patients on 10^+^ meds prescribed an Antipsychotic
  - **higher** % of older patients on 10^+^ meds prescribed a Sulfonylurea

Table 1: Proportion and number of patients age 65^+^ prescribed 10^+^ unique medications in past 12 months

|  | **# and % of patients age 65^+^ and on 10^+^ unique medications** | | **# and % of patients prescribed with at least 1 of the 4 targeted medications** | |
| --- | --- | --- | --- | --- |
|  | **My Practice** | **UTOPIAN*** | **My Practice** | **UTOPIAN*** |
| **T0** | 25 (16%) | 3% | 18 (72%) | 10 (56%) |
| **T1** | 24 (15%) | 3% | 15 (63%) | 10 (51%) |
| **T2** | 22 (13%) | 3% | 13 (59%) | 9 (50%) |
| **T3** |  |  |  |  |

* Best 10% of practices with at least 100 elderly patients

Table 2: Proportion of elderly patients on 10^+^ medications prescribed with at least one of the four targeted drugs

|  | **PPIs** | | **Benzodiazepines/**  **Z-drugs** | | **Antipsychotics** | | **Sulfonylureas** | |
| --- | --- | --- | --- | --- | --- | --- | --- | --- |
|  | **My Practice** | **UTOPIAN*** | **My Practice** | **UTOPIAN*** | **My Practice** | **UTOPIAN*** | **My Practice** | **UTOPIAN*** |
| **T0** | 11(44%) | 30% | 8 (32%) | 7% | 2 (8%) | 0% | 3 (12%) | 0% |
| **T1** | 9 (38%) | 32% | 6 (25%) | 8% | 1 (4%) | 0% | 3 (13%) | 0% |
| **T2** | 7 (32%) | 31% | 5 (23%) | 8% | 1 (5%) | 0% | 2 (9%) | 0% |
| **T3** |  |  |  |  |  |  |  |  |

* UTOPIAN practices with at least 100 patients age 65 or more

My Practice in Detail

# Date of Data Extraction

Dec 31^st^, 2019 (T2)

# Patient Inclusion Criteria

- Age 65^+^ as of the data extraction date
- Prescribed 10^+^ unique medications
- At least 2 encounters with participating provider in past 24 months
- Rostered as active in EMR

# [Confidential] Physician ID

XXX

# Number of patients who met the inclusion criteria

**22**

Table 3: Characteristics of my elderly patients with ≥10^+^ prescriptions for unique medications in past 12 months

| Pt EMR ID | # of unique meds prescribed in  past 12m | | | | At least 1 PPI prescribed in  past 12m | | | | At least 1 BZD /  Z-drug prescribed  in past 12m | | | | At least 1 AP prescribed in  past 12m | | | | At least 1 sulfonylurea prescribed in in  past 12m | | | | # of contacts in EMR in past 24m | | | |
| --- | --- | --- | --- | --- | --- | --- | --- | --- | --- | --- | --- | --- | --- | --- | --- | --- | --- | --- | --- | --- | --- | --- | --- | --- |
|  | **T0** | **T1** | **T2** | **T3** | **T0** | **T1** | **T2** | **T3** | **T0** | **T1** | **T2** | **T3** | **T0** | **T1** | **T2** | **T3** | **T0** | **T1** | **T2** | **T3** | **T0** | **T1** | **T2** | **T3** |
| 1 | 17 | 17 | 17 |  | no | no | no |  | no | no | no |  | no | no | no |  | no | no | no |  | 17 | 17 | 17 |  |
| 2 | 17 | 17 | 17 |  | **yes** | **yes** | **yes** |  | no | no | no |  | no | no | no |  | no | no | no |  | 28 | 28 | 28 |  |
| 3 | 15 | 15 | 15 |  | no | no | no |  | no | no | no |  | no | no | no |  | **yes** | **yes** | no |  | 27 | 27 | 27 |  |
| 4 | 14 | 14 | 14 |  | **yes** | **yes** | **yes** |  | no | no | no |  | no | no | no |  | no | no | no |  | 15 | 15 | 15 |  |
| 5 | 14 | 14 | 14 |  | **yes** | **yes** | no |  | no | no | no |  | no | no | no |  | **yes** | **yes** | **yes** |  | 18 | 18 | 18 |  |
| 6 | 13 | 13 | 13 |  | no | no | no |  | no | no | no |  | no | no | no |  | no | no | no |  | 20 | 20 | 20 |  |
| 7 | 13 | 13 | 13 |  | no | no | no |  | **yes** | **yes** | **yes** |  | no | no | no |  | no | no | no |  | 7 | 7 | 7 |  |
| 8 | 13 | 13 | 13 |  | no | no | no |  | **yes** | **yes** | **yes** |  | no | no | no |  | no | no | no |  | 4 | 4 | 4 |  |
| 9 | 12 | 12 | 12 |  | no | no | no |  | no | no | no |  | no | no | no |  | no | no | no |  | 18 | 18 | 18 |  |
| 10 | 12 | 12 | 12 |  | no | no | no |  | no | no | no |  | no | no | no |  | no | no | no |  | 19 | 19 | 19 |  |
| 11 | 12 | 12 | 12 |  | no | no | no |  | **yes** | **yes** | **yes** |  | no | no | no |  | no | no | no |  | 21 | 21 | 21 |  |
| 12 | 12 | 12 | 12 |  | **yes** | **yes** | **yes** |  | no | no | no |  | no | no | no |  | **yes** | **yes** | **yes** |  | 24 | 24 | 24 |  |
| 13 | 12 | 12 | 12 |  | no | no | no |  | **yes** | **yes** | **yes** |  | no | no | no |  | no | no | no |  | 28 | 28 | 28 |  |
| 14 | 12 | 12 | 12 |  | **yes** | **yes** | **yes** |  | no | no | no |  | **yes** | **yes** | **yes** |  | no | no | no |  | 14 | 14 | 14 |  |
| 15 | 12 | 12 | 12 |  | no | no | no |  | **yes** | **yes** | **yes** |  | no | no | no |  | no | no | no |  | 24 | 24 | 24 |  |
| 16 | 11 | 11 | 11 |  | no | no | no |  | no | no | no |  | no | no | no |  | no | no | no |  | 34 | 34 | 34 |  |
| 17 | 10 | 10 | 10 |  | **yes** | **yes** | **yes** |  | no | no | no |  | **yes** | no | no |  | no | no | no |  | 15 | 15 | 15 |  |
| 18 | 10 | 10 | 10 |  | **yes** | **yes** | **yes** |  | no | no | no |  | no | no | no |  | no | no | no |  | 20 | 20 | 20 |  |
| 19 | 10 | 10 | 10 |  | no | no | no |  | no | no | no |  | no | no | no |  | no | no | no |  | 20 | 20 | 20 |  |
| 20 | 10 | 10 | 10 |  | **yes** | **yes** | **yes** |  | **yes** | no | no |  | no | no | no |  | no | no | no |  | 17 | 17 | 17 |  |
| 21 | 10 | 10 | 10 |  | no | no | no |  | **yes** | no | no |  | no | no | no |  | no | no | no |  | 23 | 23 | 23 |  |
| 22 | 10 | 10 | 10 |  | no | no | no |  | **yes** | **yes** | no |  | no | no | no |  | no | no | no |  | 21 | 21 | 21 |  |
| 23 | 10 | 10 |  |  | **yes** | no |  |  | no | no |  |  | no | no |  |  | no | no |  |  | 15 | 15 |  |  |
| 24 | 10 | 10 |  |  | **yes** | **yes** |  |  | no | no |  |  | no | no |  |  | no | no |  |  | 11 | 11 |  |  |
| 25 | 10 |  |  |  | **yes** |  |  |  | no |  |  |  | no |  |  |  | no |  |  |  | 13 |  |  |  |

**Ten elderly patients in your practice have been prescribed 10^+^ unique medications and have at least 1 prescription for 1 of the medications targeted.**

- Would reviewing the charts of these patients help?
- Could a conversation with some of these elders be worthwhile?
- What might be some initial steps you could take? For which medications and which patients?
- How would you plan this?

| Which Medications? | Which Patients? |
| --- | --- |
| PPIs | 7 patients |
| Benzodiazepines | 5 patients |
| Antipsychotics | 1 patient |
| Sulfonylureas | 2 patients |

1. CIHI. 2014. Drug use among seniors on public drug programs in Canada, 2012. Retrieved from: <https://secure.cihi.ca/free_products/Drug_Use_in_Seniors_on_Public_Drug_Programs_2012_EN_web.pdf> [↑](#footnote-ref-1)
2. All data are based on practice EMR derived from CPCSSN/UTOPIAN Data Safe Haven. [↑](#footnote-ref-2)
