# Supplementary material for: Improving care for elderly patients living with polypharmacy: protocol for a pragmatic cluster randomized trial in community-based primary care practices in Canada
Source: Implement Sci. 2019 Jun 6;14:55. doi: 10.1186/s13012-019-0904-4 (PMC6551894; doi:10.1186/s13012-019-0904-4)
Supplement: Supplementary file 3 — ATC codes used to determine PIP classes in this study (DOCX 22 kb) [file 13012_2019_904_MOESM3_ESM.docx]

Appendix xx: ATC Codes for Targeted Classes of Medications

| **Medication Classes** | **ATC Code** | **Sub-Code** | **Substance Name** |
| --- | --- | --- | --- |
| Proton pump inhibitors | A02BC | A02BC01 | [omeprazole](https://www.whocc.no/atc_ddd_index/?code=A02BC01&showdescription=yes) |
|  |  | A02BC02 | [pantoprazole](https://www.whocc.no/atc_ddd_index/?code=A02BC02&showdescription=yes) |
|  |  | A02BC03 | [lansoprazole](https://www.whocc.no/atc_ddd_index/?code=A02BC03&showdescription=yes) |
|  |  | A02BC04 | [rabeprazole](https://www.whocc.no/atc_ddd_index/?code=A02BC04&showdescription=yes) |
|  |  | A02BC05 | [esomeprazole](https://www.whocc.no/atc_ddd_index/?code=A02BC05&showdescription=yes) |
|  |  | A02BC06 | [dexlansoprazole](https://www.whocc.no/atc_ddd_index/?code=A02BC06&showdescription=yes) |
|  |  | A02BC07 | [dexrabeprazole](https://www.whocc.no/atc_ddd_index/?code=A02BC07&showdescription=yes) |
|  |  | A02BC53 | [lansoprazole, combinations](https://www.whocc.no/atc_ddd_index/?code=A02BC53&showdescription=yes) |
|  |  | A02BC54 | [rabeprazole, combinations](https://www.whocc.no/atc_ddd_index/?code=A02BC54&showdescription=yes) |
| Hypoglycemic sulfonylureas | A10BB | A10BB01 | [glibenclamide](https://www.whocc.no/atc_ddd_index/?code=A10BB01&showdescription=yes) |
|  |  | A10BB02 | [chlorpropamide](https://www.whocc.no/atc_ddd_index/?code=A10BB02&showdescription=yes) |
|  |  | A10BB03 | [tolbutamide](https://www.whocc.no/atc_ddd_index/?code=A10BB03&showdescription=yes) |
|  |  | A10BB04 | [glibornuride](https://www.whocc.no/atc_ddd_index/?code=A10BB04&showdescription=yes) |
|  |  | A10BB05 | [tolazamide](https://www.whocc.no/atc_ddd_index/?code=A10BB05&showdescription=yes) |
|  |  | A10BB06 | [carbutamide](https://www.whocc.no/atc_ddd_index/?code=A10BB06&showdescription=yes) |
|  |  | A10BB07 | [glipizide](https://www.whocc.no/atc_ddd_index/?code=A10BB07&showdescription=yes) |
|  |  | A10BB08 | [gliquidone](https://www.whocc.no/atc_ddd_index/?code=A10BB08&showdescription=yes) |
|  |  | A10BB09 | [gliclazide](https://www.whocc.no/atc_ddd_index/?code=A10BB09&showdescription=yes) |
|  |  | A10BB10 | [metahexamide](https://www.whocc.no/atc_ddd_index/?code=A10BB10&showdescription=yes) |
|  |  | A10BB11 | [glisoxepide](https://www.whocc.no/atc_ddd_index/?code=A10BB11&showdescription=yes) |
|  |  | A10BB12 | [glimepiride](https://www.whocc.no/atc_ddd_index/?code=A10BB12&showdescription=yes) |
|  |  | A10BB31 | [acetohexamide](https://www.whocc.no/atc_ddd_index/?code=A10BB31&showdescription=yes) |
| Benzodiazepines or Z drugs | N05BA | N05BA01 | [diazepam](https://www.whocc.no/atc_ddd_index/?code=N05BA01&showdescription=yes) |
|  |  | N05BA02 | [chlordiazepoxide](https://www.whocc.no/atc_ddd_index/?code=N05BA02&showdescription=yes) |
|  |  | N05BA03 | [medazepam](https://www.whocc.no/atc_ddd_index/?code=N05BA03&showdescription=yes) |
|  |  | N05BA04 | [oxazepam](https://www.whocc.no/atc_ddd_index/?code=N05BA04&showdescription=yes) |
|  |  | N05BA05 | [potassium clorazepate](https://www.whocc.no/atc_ddd_index/?code=N05BA05&showdescription=yes) |
|  |  | N05BA06 | [lorazepam](https://www.whocc.no/atc_ddd_index/?code=N05BA06&showdescription=yes) |
|  |  | N05BA07 | [adinazolam](https://www.whocc.no/atc_ddd_index/?code=N05BA07&showdescription=yes) |
|  |  | N05BA08 | [bromazepam](https://www.whocc.no/atc_ddd_index/?code=N05BA08&showdescription=yes) |
|  |  | N05BA09 | [clobazam](https://www.whocc.no/atc_ddd_index/?code=N05BA09&showdescription=yes) |
|  |  | N05BA10 | [ketazolam](https://www.whocc.no/atc_ddd_index/?code=N05BA10&showdescription=yes) |
|  |  | N05BA11 | [prazepam](https://www.whocc.no/atc_ddd_index/?code=N05BA11&showdescription=yes) |
|  |  | N05BA12 | [alprazolam](https://www.whocc.no/atc_ddd_index/?code=N05BA12&showdescription=yes) |
|  |  | N05BA13 | [halazepam](https://www.whocc.no/atc_ddd_index/?code=N05BA13&showdescription=yes) |
|  |  | N05BA14 | [pinazepam](https://www.whocc.no/atc_ddd_index/?code=N05BA14&showdescription=yes) |
|  |  | N05BA15 | [camazepam](https://www.whocc.no/atc_ddd_index/?code=N05BA15&showdescription=yes) |
|  |  | N05BA16 | [nordazepam](https://www.whocc.no/atc_ddd_index/?code=N05BA16&showdescription=yes) |
|  |  | N05BA17 | [fludiazepam](https://www.whocc.no/atc_ddd_index/?code=N05BA17&showdescription=yes) |
|  |  | N05BA18 | [ethyl loflazepate](https://www.whocc.no/atc_ddd_index/?code=N05BA18&showdescription=yes) |
|  |  | N05BA19 | [etizolam](https://www.whocc.no/atc_ddd_index/?code=N05BA19&showdescription=yes) |
|  |  | N05BA21 | [clotiazepam](https://www.whocc.no/atc_ddd_index/?code=N05BA21&showdescription=yes) |
|  |  | N05BA22 | [cloxazolam](https://www.whocc.no/atc_ddd_index/?code=N05BA22&showdescription=yes) |
|  |  | N05BA23 | [tofisopam](https://www.whocc.no/atc_ddd_index/?code=N05BA23&showdescription=yes) |
|  |  | N05BA24 | [bentazepam](https://www.whocc.no/atc_ddd_index/?code=N05BA24&showdescription=yes) |
|  |  | N05BA56 | [lorazepam, combinations](https://www.whocc.no/atc_ddd_index/?code=N05BA56&showdescription=yes) |
|  | N05CD | N05CD01 | [flurazepam](https://www.whocc.no/atc_ddd_index/?code=N05CD01&showdescription=yes) |
|  |  | N05CD02 | [nitrazepam](https://www.whocc.no/atc_ddd_index/?code=N05CD02&showdescription=yes) |
|  |  | N05CD03 | [flunitrazepam](https://www.whocc.no/atc_ddd_index/?code=N05CD03&showdescription=yes) |
|  |  | N05CD04 | [estazolam](https://www.whocc.no/atc_ddd_index/?code=N05CD04&showdescription=yes) |
|  |  | N05CD05 | [triazolam](https://www.whocc.no/atc_ddd_index/?code=N05CD05&showdescription=yes) |
|  |  | N05CD06 | [lormetazepam](https://www.whocc.no/atc_ddd_index/?code=N05CD06&showdescription=yes) |
|  |  | N05CD07 | [temazepam](https://www.whocc.no/atc_ddd_index/?code=N05CD07&showdescription=yes) |
|  |  | N05CD08 | [midazolam](https://www.whocc.no/atc_ddd_index/?code=N05CD08&showdescription=yes) |
|  |  | N05CD09 | [brotizolam](https://www.whocc.no/atc_ddd_index/?code=N05CD09&showdescription=yes) |
|  |  | N05CD10 | [quazepam](https://www.whocc.no/atc_ddd_index/?code=N05CD10&showdescription=yes) |
|  |  | N05CD11 | [loprazolam](https://www.whocc.no/atc_ddd_index/?code=N05CD11&showdescription=yes) |
|  |  | N05CD12 | [doxefazepam](https://www.whocc.no/atc_ddd_index/?code=N05CD12&showdescription=yes) |
|  |  | N05CD13 | [cinolazepam](https://www.whocc.no/atc_ddd_index/?code=N05CD13&showdescription=yes) |
|  | N05CF | N05CF01 | [zopiclone](https://www.whocc.no/atc_ddd_index/?code=N05CF01&showdescription=yes) |
|  |  | N05CF02 | [zolpidem](https://www.whocc.no/atc_ddd_index/?code=N05CF02&showdescription=yes) |
|  |  | N05CF03 | [zaleplon](https://www.whocc.no/atc_ddd_index/?code=N05CF03&showdescription=yes) |
|  |  | N05CF04 | [eszopiclone](https://www.whocc.no/atc_ddd_index/?code=N05CF04&showdescription=yes) |
| Antipsychotics | N05A | N05AA01 | [chlorpromazine](https://www.whocc.no/atc_ddd_index/?code=N05AA01&showdescription=yes) |
|  |  | N05AA02 | [levomepromazine](https://www.whocc.no/atc_ddd_index/?code=N05AA02&showdescription=yes) |
|  |  | N05AA03 | [promazine](https://www.whocc.no/atc_ddd_index/?code=N05AA03&showdescription=yes) |
|  |  | N05AA04 | [acepromazine](https://www.whocc.no/atc_ddd_index/?code=N05AA04&showdescription=yes) |
|  |  | N05AA05 | [triflupromazine](https://www.whocc.no/atc_ddd_index/?code=N05AA05&showdescription=yes) |
|  |  | N05AA06 | [cyamemazine](https://www.whocc.no/atc_ddd_index/?code=N05AA06&showdescription=yes) |
|  |  | N05AA07 | [chlorproethazine](https://www.whocc.no/atc_ddd_index/?code=N05AA07&showdescription=yes) |
|  |  | N05AB01 | [dixyrazine](https://www.whocc.no/atc_ddd_index/?code=N05AB01&showdescription=yes) |
|  |  | N05AB02 | [fluphenazine](https://www.whocc.no/atc_ddd_index/?code=N05AB02&showdescription=yes) |
|  |  | N05AB03 | [perphenazine](https://www.whocc.no/atc_ddd_index/?code=N05AB03&showdescription=yes) |
|  |  | N05AB04 | [prochlorperazine](https://www.whocc.no/atc_ddd_index/?code=N05AB04&showdescription=yes) |
|  |  | N05AB05 | [thiopropazate](https://www.whocc.no/atc_ddd_index/?code=N05AB05&showdescription=yes) |
|  |  | N05AB06 | [trifluoperazine](https://www.whocc.no/atc_ddd_index/?code=N05AB06&showdescription=yes) |
|  |  | N05AB07 | [acetophenazine](https://www.whocc.no/atc_ddd_index/?code=N05AB07&showdescription=yes) |
|  |  | N05AB08 | [thioproperazine](https://www.whocc.no/atc_ddd_index/?code=N05AB08&showdescription=yes) |
|  |  | N05AB09 | [butaperazine](https://www.whocc.no/atc_ddd_index/?code=N05AB09&showdescription=yes) |
|  |  | N05AB10 | [perazine](https://www.whocc.no/atc_ddd_index/?code=N05AB10&showdescription=yes) |
|  |  | N05AC01 | [periciazine](https://www.whocc.no/atc_ddd_index/?code=N05AC01&showdescription=yes) |
|  |  | N05AC02 | [thioridazine](https://www.whocc.no/atc_ddd_index/?code=N05AC02&showdescription=yes) |
|  |  | N05AC03 | [mesoridazine](https://www.whocc.no/atc_ddd_index/?code=N05AC03&showdescription=yes) |
|  |  | N05AC04 | [pipotiazine](https://www.whocc.no/atc_ddd_index/?code=N05AC04&showdescription=yes) |
|  |  | N05AD01 | [haloperidol](https://www.whocc.no/atc_ddd_index/?code=N05AD01&showdescription=yes) |
|  |  | N05AD02 | [trifluperidol](https://www.whocc.no/atc_ddd_index/?code=N05AD02&showdescription=yes) |
|  |  | N05AD03 | [melperone](https://www.whocc.no/atc_ddd_index/?code=N05AD03&showdescription=yes) |
|  |  | N05AD04 | [moperone](https://www.whocc.no/atc_ddd_index/?code=N05AD04&showdescription=yes) |
|  |  | N05AD05 | [pipamperone](https://www.whocc.no/atc_ddd_index/?code=N05AD05&showdescription=yes) |
|  |  | N05AD06 | [bromperidol](https://www.whocc.no/atc_ddd_index/?code=N05AD06&showdescription=yes) |
|  |  | N05AD07 | [benperidol](https://www.whocc.no/atc_ddd_index/?code=N05AD07&showdescription=yes) |
|  |  | N05AD08 | [droperidol](https://www.whocc.no/atc_ddd_index/?code=N05AD08&showdescription=yes) |
|  |  | N05AD09 | [fluanisone](https://www.whocc.no/atc_ddd_index/?code=N05AD09&showdescription=yes) |
|  |  | N05AE01 | [oxypertine](https://www.whocc.no/atc_ddd_index/?code=N05AE01&showdescription=yes) |
|  |  | N05AE02 | [molindone](https://www.whocc.no/atc_ddd_index/?code=N05AE02&showdescription=yes) |
|  |  | N05AE03 | [sertindole](https://www.whocc.no/atc_ddd_index/?code=N05AE03&showdescription=yes) |
|  |  | N05AE04 | [ziprasidone](https://www.whocc.no/atc_ddd_index/?code=N05AE04&showdescription=yes) |
|  |  | N05AE05 | [lurasidone](https://www.whocc.no/atc_ddd_index/?code=N05AE05&showdescription=yes) |
|  |  | N05AF01 | [flupentixol](https://www.whocc.no/atc_ddd_index/?code=N05AF01&showdescription=yes) |
|  |  | N05AF02 | [clopenthixol](https://www.whocc.no/atc_ddd_index/?code=N05AF02&showdescription=yes) |
|  |  | N05AF03 | [chlorprothixene](https://www.whocc.no/atc_ddd_index/?code=N05AF03&showdescription=yes) |
|  |  | N05AF04 | [tiotixene](https://www.whocc.no/atc_ddd_index/?code=N05AF04&showdescription=yes) |
|  |  | N05AF05 | [zuclopenthixol](https://www.whocc.no/atc_ddd_index/?code=N05AF05&showdescription=yes) |
|  |  | N05AG01 | [fluspirilene](https://www.whocc.no/atc_ddd_index/?code=N05AG01&showdescription=yes) |
|  |  | N05AG02 | [pimozide](https://www.whocc.no/atc_ddd_index/?code=N05AG02&showdescription=yes) |
|  |  | N05AG03 | [penfluridol](https://www.whocc.no/atc_ddd_index/?code=N05AG03&showdescription=yes) |
|  |  | N05AH01 | [loxapine](https://www.whocc.no/atc_ddd_index/?code=N05AH01&showdescription=yes) |
|  |  | N05AH02 | [clozapine](https://www.whocc.no/atc_ddd_index/?code=N05AH02&showdescription=yes) |
|  |  | N05AH03 | [olanzapine](https://www.whocc.no/atc_ddd_index/?code=N05AH03&showdescription=yes) |
|  |  | N05AH04 | [quetiapine](https://www.whocc.no/atc_ddd_index/?code=N05AH04&showdescription=yes) |
|  |  | N05AH05 | [asenapine](https://www.whocc.no/atc_ddd_index/?code=N05AH05&showdescription=yes) |
|  |  | N05AH06 | [clotiapine](https://www.whocc.no/atc_ddd_index/?code=N05AH06&showdescription=yes) |
|  |  | N05AL01 | [sulpiride](https://www.whocc.no/atc_ddd_index/?code=N05AL01&showdescription=yes) |
|  |  | N05AL02 | [sultopride](https://www.whocc.no/atc_ddd_index/?code=N05AL02&showdescription=yes) |
|  |  | N05AL03 | [tiapride](https://www.whocc.no/atc_ddd_index/?code=N05AL03&showdescription=yes) |
|  |  | N05AL04 | [remoxipride](https://www.whocc.no/atc_ddd_index/?code=N05AL04&showdescription=yes) |
|  |  | N05AL05 | [amisulpride](https://www.whocc.no/atc_ddd_index/?code=N05AL05&showdescription=yes) |
|  |  | N05AL06 | [veralipride](https://www.whocc.no/atc_ddd_index/?code=N05AL06&showdescription=yes) |
|  |  | N05AL07 | [levosulpiride](https://www.whocc.no/atc_ddd_index/?code=N05AL07&showdescription=yes) |
|  |  | N05AN01 | [lithium](https://www.whocc.no/atc_ddd_index/?code=N05AN01&showdescription=yes) |
|  |  | N05AX07 | [prothipendyl](https://www.whocc.no/atc_ddd_index/?code=N05AX07&showdescription=yes) |
|  |  | N05AX08 | [risperidone](https://www.whocc.no/atc_ddd_index/?code=N05AX08&showdescription=yes) |
|  |  | N05AX10 | [mosapramine](https://www.whocc.no/atc_ddd_index/?code=N05AX10&showdescription=yes) |
|  |  | N05AX11 | [zotepine](https://www.whocc.no/atc_ddd_index/?code=N05AX11&showdescription=yes) |
|  |  | N05AX12 | [aripiprazole](https://www.whocc.no/atc_ddd_index/?code=N05AX12&showdescription=yes) |
|  |  | N05AX13 | [paliperidone](https://www.whocc.no/atc_ddd_index/?code=N05AX13&showdescription=yes) |
|  |  | N05AX14 | [iloperidone](https://www.whocc.no/atc_ddd_index/?code=N05AX14&showdescription=yes) |
|  |  | N05AX15 | [cariprazine](https://www.whocc.no/atc_ddd_index/?code=N05AX15&showdescription=yes) |
|  |  | N05AX16 | [brexpiprazole](https://www.whocc.no/atc_ddd_index/?code=N05AX16&showdescription=yes) |
|  |  | N05AX17 | [pimavanserin](https://www.whocc.no/atc_ddd_index/?code=N05AX17&showdescription=yes) |
