# Supplementary material for: Improving care for elderly patients living with polypharmacy: protocol for a pragmatic cluster randomized trial in community-based primary care practices in Canada
Source: Implement Sci. 2019 Jun 6;14:55. doi: 10.1186/s13012-019-0904-4 (PMC6551894; doi:10.1186/s13012-019-0904-4)
Supplement: Supplementary file 4 — EMR data extraction (PDF 553 kb) [file 13012_2019_904_MOESM4_ESM.pdf]

CIHR IRSC Canadian Institutes of Health Research / Instituts de recherche en santé du Canada · NORTH YORK GENERAL · Family & Community Medicine UNIVERSITY OF TORONTO · UTOPIAN · Improvement Education Innovation · Quality & Innovation Program

A Research and QI Collaboration Supporting Practices in Improving Care for Complex Elderly Patients

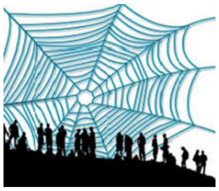

SPIDER: A Structured Process Informed by Data, Evidence and Research.

Learning Collaborative Launch
September 14, 2018
DFCM (Rooms 303/365), 500 University Avenue, Toronto

Time	Minutes	Activities	Lead
08:30 to 08:55	25 min	**Welcome**<br>– Introductions<br>– Launch overview<br>– **System Thinking** - 'Clap Focus'	Michelle Greiver, Trish O'Brien & Jeremy Rezmovitz
08:55 to 09:25	30 min	**What is SPIDER?**<br>– The research project<br>– The evidence base	Michelle Greiver
09:25 to 10:00	35 min	**The Quality Improvement Approach**<br>– **Collaboration** - 'Complete the Picture'<br>– Learning collaborative design<br>– Patient engagement	Trish O'Brien, Jeremy Rezmovitz & Teams
10:00 to 10:20	20 min	Break	
10:20 to 11:05	45 min	**Decision-making with Data to Improve Care**<br>– Reflecting on practice-level data<br>– Deciding on action	Noah Ivers
11:05 to 12:00	55 min	**The Quality Improvement Approach (cont.)**<br>– Team engagement<br>– QI Coach - 'improvement enabler'<br>– QI Curriculum<br>– Improvement Pathway	Trish O'Brien, Christina Southey, Jeremy Rezmovitz & Teams
12:00 to 13:00	60 min	**System Thinking** - 'Clap Focus'<br>Lunch	

Time	Minutes	Activities	Lead
13:00 to 13:30	30 min	**Working together for improvement**<br>'Group Think'	Jeremy Rezmovitz & Teams
13:30 to 14:25	55 min	**Charting the Pathway to Improvement**<br>- Initiating the QI Project Charter	Trish O'Brien, Michelle Greiver & Jeremy Rezmovitz
14:25 to 14:45	20 min	Break	
14:45 to 15:00	15 min	**Reflecting on what matters**<br>– 'Mad Tea Party'	Trish O'Brien & Jeremy Rezmovitz
15:00 to 15:45	45 min	**Charting the Pathway to Improvement (Cont'd)**<br>– Incorporating the evidence<br>– Project Charter planning	Trish O'Brien & Michelle Greiver
15:45 to 16:00	15 min	**Wrap-up**<br>– Evaluation	Trish O'Brien, Christina Southey & Teams
